# Identification and functional characterisation of a novel N-cyanoamidine neonicotinoid metabolising cytochrome P450, CYP9Q6, from the buff-tailed bumblebee *Bombus terrestris*

**DOI:** 10.1016/j.ibmb.2019.05.006

**Published:** 2019-08

**Authors:** Bartlomiej J. Troczka, Rafael A. Homem, Rebecca Reid, Katherine Beadle, Maxie Kohler, Marion Zaworra, Linda M. Field, Martin S. Williamson, Ralf Nauen, Chris Bass, T.G Emyr Davies

**Affiliations:** a Biointeractions and Crop Protection Department, Rothamsted Research, West Common, Harpenden, AL5 2JQ, UK; b College of Life and Environmental Sciences, Biosciences, University of Exeter, Penryn Campus, Penryn, Cornwall, UK; c Bayer AG, Crop Science Division, R&D, Alfred Nobel-Strasse 50, 40789, Monheim, Germany

**Keywords:** Bumblebee, Neonicotinoids, Cytochrome P450, Metabolism

## Abstract

Recent work has shown that two bumblebee (*Bombus terrestris*) cytochrome P450s of the CYP9Q subfamily, CYP9Q4 and CYP9Q5, are important biochemical determinants of sensitivity to neonicotinoid insecticides. Here, we report the characterisation of a third P450 gene *CYP9Q6*, previously mis-annotated in the genome of *B. terrestris,* encoding an enzyme that metabolises the *N*-cyanoamidine neonicotinoids thiacloprid and acetamiprid with high efficiency. The genomic location and complete ORF of *CYP9Q6* was corroborated by PCR and its metabolic activity characterised *in vitro* by expression in an insect cell line. CYP9Q6 metabolises both thiacloprid and acetamiprid more rapidly than the previously reported CYP9Q4 and CYP9Q5. We further demonstrate a direct, *in vivo* correlation between the expression of the CYP9Q6 enzyme in transgenic *Drosophila melanogaster* and an increased tolerance to thiacloprid and acetamiprid. We conclude that CYP9Q6 is an efficient metaboliser of *N*-cyanoamidine neonicotinoids and likely plays a key role in the high tolerance of *B. terrestris* to these insecticides.

## Highlights

• A cytochrome P450 gene CYP9Q6 is identified as a potential N-cyanoamidine neonicotinoid metaboliser in *Bombus terrestris*.
• CYP9Q6 efficiently metabolized N-cyanoamidine neonicotinoid insecticides when expressed *in vitro* in insect cell lines.
• Transgenic *D. melanogaster* expressing CYP9Q6 *in vivo* had increased tolerance to N-cyanoamidine neonicotinoids.
• Amino acid substitutions in the P450 active site may be important determinants of binding and metabolism of these compounds.

## Graphical abstract

**Figure:**
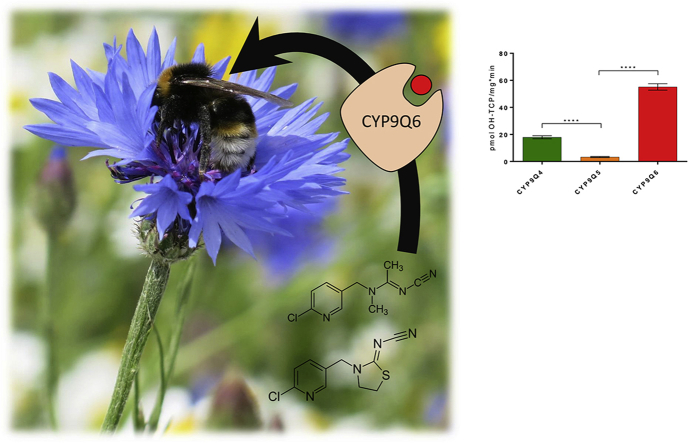

## 1 Introduction

The buff-tailed bumblebee, *Bombus terrestris* (Hymenoptera: Apidae) is one of the most abundant bumblebee species in Europe and an important pollinator of wild plants and crops. Domesticated colonies are used for managed pollination of crops in orchards and greenhouses and are one of the main commercial alternatives to the western honey bee, *Apis mellifera* (Goulson, 2010; Velthuis and van Doorn, 2006). Apart from their commercial importance bumblebees are also used as a model species for bee population genetics and the study of insect behaviour (Woodard et al., 2015).

The effect of xenobiotics on the health of bee pollinators has been the focus of considerable recent research, particularly in relation to the extensive use of synthetic insecticides in agriculture (Goulson et al., 2015). The effect of insecticidal exposure on the overall fitness of individuals and colonies of this species is still a topic of active investigation (Potts et al., 2018; Wu-Smart and Spivak, 2018). However, concerns over the effects of three *N*-nitroguanidine neonicotinoid insecticides, imidacloprid, thiamethoxam and clothianidin on bee pollinators, including *B. terrestris,* has led the European Union to ban these compounds for use on field crops (Cressey, 2017). Due to their high acute intrinsic toxicity to *B. terrestris* these three compounds are all classified as ‘highly toxic’ according to the categories delineated by the U.S. Environmental Protection Agency (Manjon et al., 2018). In contrast *B. terrestris* exhibits a marked tolerance to *N*-cyanoamidine neonicotinoids, with thiacloprid classified as ‘practically non-toxic’ in acute contact bioassays (U.S.E.P. Agency, 2014; Manjon et al., 2018). Similar differences have been described for the toxicity of the *N*-nitroguanidines and *N*-cyanoamidines to the western honey bee, *A. mellifera* (Manjon et al., 2018), and the solitary red mason bee, *Osmia bicornis* (Beadle et al., 2019).

Recent research has provided a biochemical explanation for this observed differential sensitivity and shown that, in all three bee species, P450 enzymes from the CYP9 family efficiently detoxify *N*-cyanoamidine compounds but have little activity against *N*-nitroguanidine compounds, thus acting as molecular determinants of bee sensitivity to neonicotinoids (Beadle et al., 2019; Manjon et al., 2018). In *B. terrestris* two P450s, CYP9Q4 and CYP9Q5, were previously shown to efficiently metabolise the *N*-cyanoamidine neonicotinoids thiacloprid and acetamiprid; however their overall combined activity in enzyme assays was significantly lower than that of CYP9Q3, the primary P450 metabolising these neonicotinoids in *A. mellifera*, leading us to speculate that additional P450s may play a role in neonicotinoid metabolism in bumblebees (Manjon et al., 2018). Here we report the identification and functional characterisation of a novel CYP9Q from *B. terrestris*, CYP9Q6, and demonstrate it to be an efficient detoxifier of *N*-cyanoamidine neonicotinoids.

## 2 Methods

### 2.1 Insects and chemicals

Worker bees used for the experiments came from *Bombus terrestris* commercial colonies (standard hives) supplied by Biobest (Belgium). Colonies were kept in the dark at 27±1 °C, 50% relative humidity. All chemicals were supplied by Sigma-Aldrich unless stated otherwise.

### 2.2 Preparation of nucleic acids

DNA and RNA extractions were done on single adult worker bees. DNA was extracted using the E.Z.N.A. Insect DNA kit (Omega-BioTek, USA) following the manufacture's protocol. Total RNA was extracted using Triazol reagent (ThermoScientific, USA) following the manufacturer's protocol. 4  μg of RNA was used for cDNA synthesis using SuperScript^®^ III reverse transcriptase (Life Technologies, USA) with Oligo_d_T_(15)_ primer (Promega, USA).

### 2.3 RT-PCR, sub-cloning and sequencing

PCR was performed using *pfu* polymerase (Promega, USA) in 25 μl reactions containing; 1  μl of cDNA or genomic DNA template, 10 pmol of each forward and reverse primer (see Supplementary Table 1 for primer details), and 1.5U of enzyme. Cycling conditions comprised an initial denaturation at 95 °C for 3 min, followed by 35 cycles of 95 °C for 20s, 55 °C for 30s, 72 °C for 4 min, with a final extension step at 72 °C for 5 min. PCR amplicons were separated using 1% w/v TAE gel electrophoresis, and products of the expected size were excised from the gel and recovered using Qiaquick gel extraction kit (Qiagen, Germany), following the manufacturer's protocol. PCR products were sequenced directly using Eurofins value read service (Germany). Bands which represented full-length ORF were further ligated into the pJET 1.2 vector of GeneJET PCR cloning kit (ThermoScientific, USA), and transformed into XL-1 blue cells (Agilent, USA). Plasmid preps were prepared using a GeneJET mini-prep kit (ThermoScientific, USA), following the manufacturer's protocol.

### 2.4 Tissue-specific qPCR

Bumblebees were dissected in PBS (phosphate buffered saline) buffer, and total RNA was prepared from tissues of a single female worker as described above. Two μg of RNA was used for cDNA synthesis as described above. PCR reactions (15 μl) contained 4 μl of cDNA (diluted 1:10 v/v), 7.5 μl of SYBR^®^ Green JumpStart™ Taq ReadyMix (Sigma-Aldrich, USA), and 0.25 μM of each primer (Supplementary Table 1). Cycling conditions comprised 3 min at 95 °C, followed by 39 cycles of 95 °C for 15s, 64 °C for 15s and 60 °C for 15s. A final melt-curve step was included post-PCR (ramping from 65 to 95 °C by 0.5 °C every 5s) to confirm the absence of any non-specific amplification. The efficiency of PCR for each primer pair was assessed using a serial dilution of 100 ng–0.01 ng of cDNA. Each qPCR experiment consisted of at least 8 independent biological replicates with three technical replicates for each. Data were analysed according to the 2^−ΔΔCt^ method (Pfaffl, 2001) in MS excel 365 (Microsoft). The expression level was normalized to two validated reference genes, PLA2 (phospholipase A2) and EEF1A (Supplementary Table 1) (Hornakova et al., 2010). Data was visualised using GraphPad prism v7 (GraphPad Software, USA).

### 2.5 Baculovirus-mediated protein expression

*B. terrestris* CYP9Q6 and house fly NADPH-dependent cytochrome P450 reductase (CPR) (GenBank accession no. Q07994) were codon optimised for expression in lepidopteran cell lines, synthesised by GeneArt^®^ (Life Technologies, USA), and inserted into the pDonor221 expression vector (Invitrogen). The recombinant baculovirus DNA was synthesised *in vitro* and transfected into *Trichoplusia ni* High five™ cells using the BaculoDirect C-term expression kit (Life Technologies, USA), according to the manufacturer's protocol. The titre of the recombinant virus was determined following protocols detailed by the supplier. High Five™ cells (Life Technologies, USA), grown to a density of 2 × 10^6^ cells ml^−1,^ were co-infected with recombinant baculoviruses containing CYP9Q6 and CPR with a range of MOI (multiplicity of infection) ratios to identify the optimal conditions. Control cells were co-infected with the baculovirus containing vector with no insert (ctrl-virus) and the recombinant baculovirus expressing CPR using the same MOI ratios. Ferric citrate and δ-aminolevulinic acid hydrochloride were added to a final concentration of 0.1  mM at the time of infection and 24 h after infection, to compensate for the low levels of endogenous heme in the insect cells. Cells were harvested, washed with PBS, and microsomes prepared according to standard procedures (Schenkman and Jansson, 2006) 48 h post infection. Briefly, cells were homogenised for 30s in 0.1 M Na/K-phosphate buffer (pH 7.4), containing 1 mM EDTA and DTT, and 200 mM sucrose, using a tissue grinder, and the homogenate centrifuged for 10 min at 680 g at 4 °C. The supernatant recovered was centrifuged for 20 min at 10,000 g and the decanted supernatant centrifuged for a further 1 h at 100,000 g at 4 °C. The pellet obtained was resuspended in 0.1 M Na/K-phosphate buffer (pH 7.6) containing 1 mM EDTA and DTT, and 10% v/v glycerol, using a Dounce tissue grinder. Cytochrome P450 expression and functionality was estimated by measuring the CO-difference spectra in reduced samples on a SLM AMINCO Dual Wavelength Spectrophotometer DW 2000 (SLM instruments) and scanning from 500 nm to 400 nm (Schenkman and Jansson, 2006). The protein content of samples was determined using Bradford reagent (Sigma-Aldrich, USA) and bovine serum albumin (BSA) as a reference.

### 2.6 Insecticide metabolism and model substrate profiling

Metabolism of thiacloprid and acetamiprid were assessed by incubating recombinant P450/CPR (50μg/well) or control virus/CPR (50μg/well) with a range of substrate concentrations (200-3.1  μM) in the presence of an NADPH regeneration system (Promega, USA) at 30 ± 1 °C, shaking, for 1 h. Three replicates were performed for each data point and the total assay volume was 200 μl. The breakdown rate was assayed exactly as described above with a single concentration of substrate (200 μM). Samples incubated without NADPH served as an additional control. The reactions were terminated by the addition of 800 μl of ice-cold acetonitrile, centrifuged for 10 min at 3000 g and the supernatant subsequently analysed by tandem mass spectrometry as described previously (Zhu et al., 2010). For chromatography on a Waters Acquity HSS T3 column (2.1 × 50mm, 1.8  μm), acetonitrile/water/0.1% formic acid was used as the eluent in gradient mode. For detection and quantification in positive ion mode, the MRM transitions 253 > 186, 269 > 202 (thiacloprid, OH-thiacloprid), and 223 > 126, 209 > 126 (acetamiprid and *N*-desmethyl acetamiprid) were monitored. The peak integrals were calibrated externally against a standard calibration curve. The linear range for the quantification of neonicotinoids and their hydroxylated (thiacloprid) and *N*-desmethylated (acetamiprid) metabolites was 0.1–1000  ng ml^−1^. Recovery rates of parent compounds using microsomal fractions without NADPH were normally close to 100%. All statistical calculations and substrate turnover from two independent reactions were plotted vs. Controls and Michaelis-Menten kinetics determined using GraphPad Prism version 7 (GraphPad Software, USA).

### 2.7 Generation of transgenic *D. melanogaster*

All Drosophila strains were maintained on standard fly food (Bloomington formulation) at 25 °C, on a 12/12 h light/dark cycle and 65% relative humidity. *Cyp9Q6* was amplified from *B. terrestris* genomic DNA and cloned into the attB-carrying plasmid pUAST (pUASTattB_EF362409) using the *Eco*RI and *Xba*I restriction sites. Injection mixes containing 0.5x phosphate buffer pH 6.8 (0.05 mM sodium phosphate, 2.5 mM KCl), 300 ng/μl of the *pUAST-Cyp9Q6* construct and 200  mg l^−1^ fluorescein sodium salt were delivered into pre-blastoderm embryos of an integration strain (y w M(eGFP, vas-int, dmRFP)ZH-2A; P{CaryP}. attp40) sourced from the Fly Facility, University of Cambridge, using an inverted microscope (eclipse TieU Nikon, Japan) equipped with a 10x/0.25 lens, 10x/22 eyepiece and fluorescence illumination and a FemtoJet express micro-injector (Eppendorf, Hamburg, Germany) controlled by a motorised TransferMan NK2 micromanipulator (Eppendorf, Hamburg, Germany). Injection needles were prepared according to Miller et al. (2002). Survivors were back-crossed and F1 individuals were screened for the presence of the *white* gene, which generates flies with orange eyes. Homozygous stocks for the integration of *pUAST-Cyp9Q6* at *attP40* were generated by inter-crossing orange eyed flies and selecting for red eyed individuals. Control flies carrying an empty pUAST plasmid were generated following the same procedures described above.

### 2.8 Transgenic *D. melanogaster* bioassays

The UAS/GAL4 system was used to drive the expression of *Cyp9Q6*. Male flies, homozygous for *pUAST-Cyp9Q6* (*UAS::CYP9Q6*) or an empty *pUAST* plasmid inserted at the same genomic location (*UAS*:*empty*), were crossed to virgin females of a heat-shock inducible GAL4 driver strain (w[*];P{wmC] = GAL4-Hsp70.PB}89-2-1) to generate *UAS::CYP9Q6/* + *;Hsp::GAL4/* + and *UAS::empty/* + *;Hsp::GAL4/* + strains, respectively. Overexpression of *Cyp9Q6* in *UAS::CYP9Q6/* + *;Hsp::GAL4/* + flies was confirmed via qPCR with the *UAS::CYP9Q6* parental strain used as a control for the relative comparisons. Reactions were setup as presented in section 2.4, a single biological replicate consisted of 5 heat-shock treated adult females. Over 600 four to eight-day old female flies from each genotype (*UAS::CYP9Q6/* + *;Hsp::GAL4/* + and *UAS::empty/* + *;Hsp::GAL4/* + ) were selected and used in contact/feeding bioassays. 24 h prior to bioassays, flies were heat-shock treated three times for 30 min at 37 °C, with 1-h intervals, to induce the expression of *Cyp9Q6*. Groups of 20 flies were transferred to vials, previously coated with 100 μl of an insecticide dilution, containing 3 ml of a settled agar-sucrose solution. Each bioassay used five different insecticide concentrations, five replications per concentration and the number of dead flies was recorded after 48 h. Control mortality was assessed using vials containing agar-sucrose without insecticide. Lethal concentrations (LC_50_ values) and 95% confidence limits were calculated by probit analysis using Genstat version 16 (VSN International, UK). Statistical significance was calculated using two tailed *t*-test in GraphPadprism version 7 (GraphPad Software, USA).

## 3 Results and discussion

Phylogeny (Supplementary figure 1) of the CYP9 P450 gene family in the annotated genomes of Apis and Bombus revealed that *A. mellifera* had three complete CYP9Q genes while *B. terrestris* had only two (the previously characterised CYP9Q4 and CYP9Q5). To investigate if an additional CYP9Q gene was present in *B. terrestris* we performed an *in silico* re-analysis of P450 sequences in the *B. terrestris* genome.

### 3.1 Identification of CYP9Q6 in the genome of *B. terrestris*

**Fig. 1:**
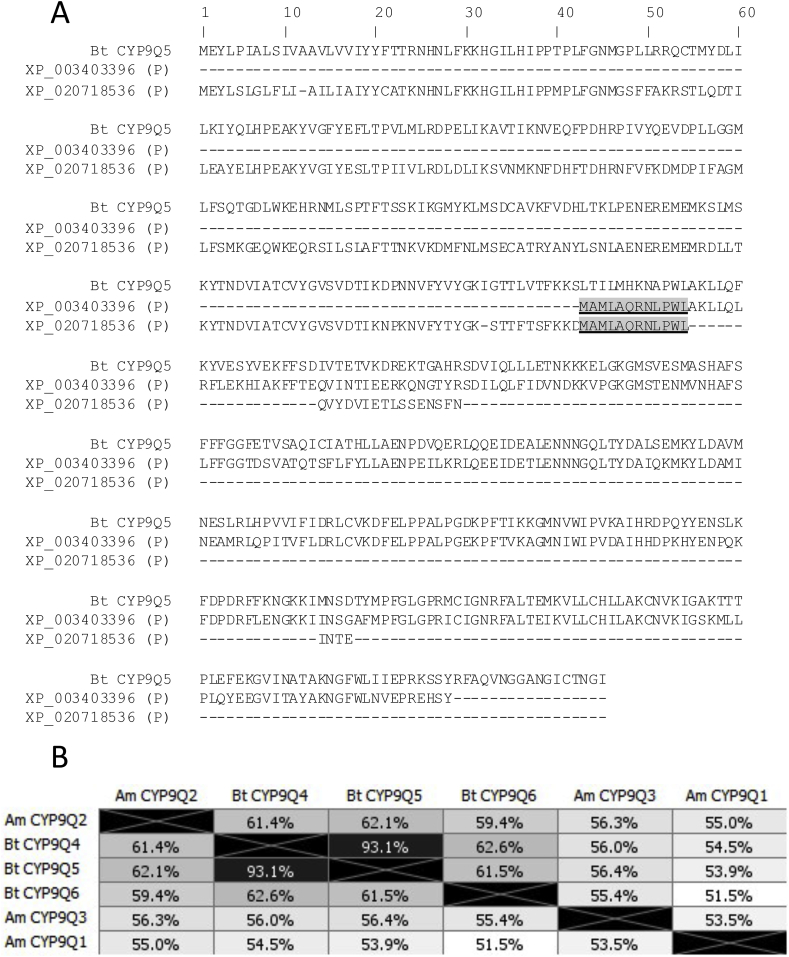
**(**A**)** Alignment of the two partial transcripts data-mined from the NCBI publicly available *Bombus terrestris* genome database with CYP9Q5. The overlapping region of the two partial transcripts is highlighted. **(B)** Amino acid identity between CYP9Qs from *B. terrestris* (Bt) and *A. mellifera* (Am)*.*

Two partial protein sequences (NCBI accession's XP_020718536.1 and XP_003403396.2) with high level homology to the CYP9Q sub-family were identified in the public protein database (Sadd et al., 2015) by BLASTp searches using *B. terrestris* CYP9Q4 and CYP9Q5 sequences as templates. Alignment of these partial transcripts to either CYP9Q4 or CYPQ5 indicated they may correspond to different regions of a novel, full-length P450 sequence (Fig. 1A). tBLASTn of the partial protein sequences against the *B. terrestris* genome identified two separate genomic locations, NCBI accession's NC_015762.1 and NW_003568850.1. Accession NC_015762.1 is a large scaffold previously identified as the location of *CYP9Q4* and *CYP9Q5*. The BLAST match was located approximately 3000bp downstream of the *CYP9Q4* gene locus, immediately upstream of a sequence gap in the assembly.

Amplification of the predicted novel ORF by RT-PCR yielded a single 1.5 Kb cDNA, confirming that the two partial transcripts are in fact part of a single expressed mRNA. PCR on genomic DNA yielded the same result, suggesting that the novel gene is intronless (as is the case for CYP9Q4 and CYP9Q5, and CYP9Q1-3 of *A. mellifera*) and forms part of the same genomic locus. Sequencing of both the genomic and reversed transcribed mRNA (cDNA) amplicons further confirmed that this is a novel, full-length P450 ORF. No premature stop codons were found in either sequence suggesting that the gene is not a pseudogene and is likely to be functional. The protein sequence of the novel gene shares 62.6% and 61.5% identity with *CYP9Q4* and *CYP9Q5* respectively and 55.4% identity with *A. mellifera CYP9Q3* (Fig. 1B), known to be a primary metaboliser of *N*-cyanoamidine neonicotinoids in this species (Manjon et al., 2018).

Previous work on the CYP9Q P450s in *A. mellifera* and *B. terrestris* revealed high levels of expression of certain members of the family in tissues with a known role in xenobiotic detoxification or sites of insecticide action, such as the Malpighian tubules and the brain. Tissue expression profiling of *CYP9Q6* mRNA transcripts revealed a slightly higher expression profile in the bumblebee brain in comparison to the midgut or Malpighian tubules (Fig. 3), although the brain expression bias is much less pronounced in comparison to CYP9Q4 (Manjon et al., 2018). These results are consistent with the high expression of *A. mellifera* CYP9Q3 in the brain (Manjon et al., 2018), suggesting that *B. terrestris* CYP9Q6 may play a similar functional role in protecting this sensitive tissue from xenobiotics that cross the blood-brain barrier.

**Fig. 2:**
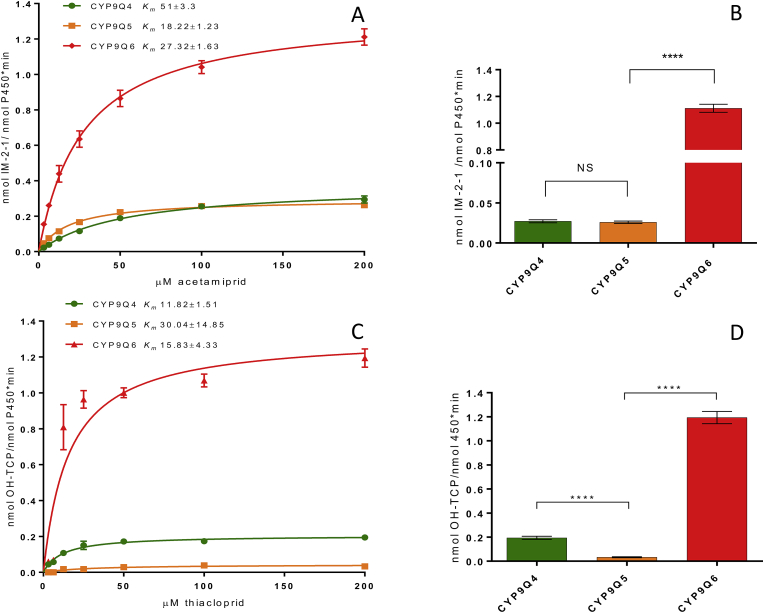
(A and C) Kinetics of acetamiprid and thiacloprid for the CYP9Q sub-family. Data for CYP9Q4 and Q5 was collated from Manjon et al. (Manjon et al., 2018). N = 3, K_m_ values shown on graph ±standard deviation. V_max_ values for Q6 are 62.44 ± 1.21 (acetamiprid), 60.87 ± 4.72 (thiacloprid). (B and D**)** Breakdown of acetamiprid and thiacloprid by *B. terrestris* CYP9Q enzymes. CYP9Q4 and Q5 data taken from Manjon et al. (Manjon et al., 2018). Error bars represent standard deviation, N = 3. Significance is displayed above, NS- not significant, **** P < 0.0001 (One-way ANOVA with Tukey test).

**Fig. 3:**
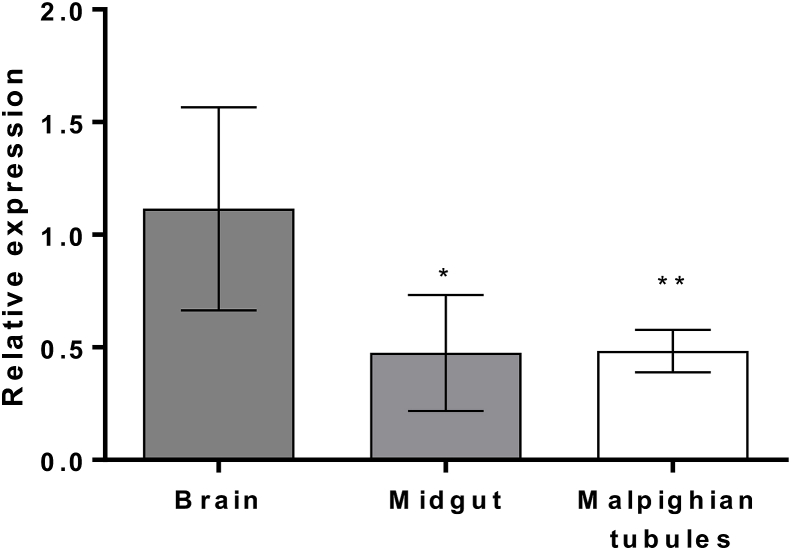
Tissue-specific expression of CYP9Q6 transcripts in adult female worker. Significant differences in expression relative to the brain are indicated with *. *P = 0.0089, **P = 0.0113 (two tailed T-test). N = 8 individual organs, error bars represent 95% confidence limits.

### 3.2 CYP9Q6 metabolises *N*-cyanoamidine neonicotinoids *in vitro*

To examine the capacity of CYP9Q6 to metabolise the *N*-cyanoamidine neonicotinoids thiacloprid and acetamiprid *in vitro,* CYP9Q6 was co-expressed with house fly cytochrome P450 reductase (CPR) in an insect cell line and microsomal preparations were incubated with each insecticide. Analysis of the resulting metabolites, by liquid chromatography tandem mass spectrometry (LC-MS/MS), revealed that recombinant CYP9Q6 readily metabolises both compounds in the presence of NADPH (Fig. 2). The K_m_ values for thiacloprid and acetamiprid were 15.83 and 27.32 μM respectively, with V_max_ values of 1.352 and 1.318 nmol product/nmol of active P450. Since the experimental setup for CYP9Q6 directly mirrored the previously reported methods for CYP9Q4 and CYPQ5, a direct comparison of the apparent rate of metabolism for CYP9Q6 and the other two CYP9Q P450s was possible. Data presented in Manjon et al. (2018) was converted from mg/ml to active nmol P450 enzyme to normalise for any difference in the expression of active P450 (see Supplementary figure 3). This showed that CYP9Q6 is 20 times more active at metabolising acetamiprid compared to either CYP9Q4 or CYP9Q5, while it is 3 (CYP9Q4) and 16 (CYP9Q5) times more active at metabolising thiacloprid. These results suggest that CYP9Q6 is the more efficient metaboliser for both compounds in *B. terrestris* (Fig. 2).

### 3.3 Model-substrate profile and catalytic site of CYP9Q6

A range of coumarin and resorufin model substrates were tested against CYP9Q6 expressed in microsomes. The enzyme does not exhibit a particularly strong affinity to any of these test compounds but did demonstrate a slight preference for simple O-alkyl coumarins and resorufins (Supplementary figure 2). This contrasts with the other two previously described *B. terrestris* CYP9Q proteins, CYP9Q4 and CYP9Q5, which only show activity against coumarins with methoxy type moieties (Manjon et al., 2018). Thus, CYP9Q6 exhibits a greater promiscuity when presented with model substrates although with a low overall turnover. In contrast, *A*. *mellifera* CYP9Q3 efficiently metabolises a broad range of coumarins (Manjon et al., 2018).

**Fig. 4:**
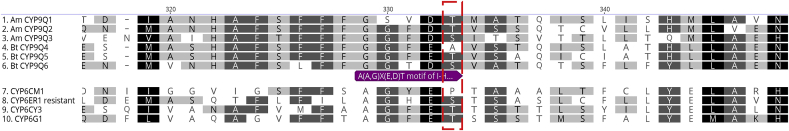
Protein alignment of the A(A,G)X(E,D)T motif of the I-Helix region of cytochrome P450s belonging to CYP9Q sub-family in *Apis mellifera* (Am) or *Bombus terrestris* (Bt) and 4 other P450s shown to metabolise neonicotinoid insecticides in insect pest species: *Bemisa tabaci* CYP6CM1, *Myzus persicae* CYP6CY3, *Drosophila melanogaster* CYP6G1 and *Nilaparvata lugens* CYP6ER1vA. The normally highly conserved threonine in the oxygen-binding motif is highlighted.

Protein alignments of CYP9Q6 with other members of the CYP9Q sub-family revealed an amino acid substitution at a highly conserved A(A,G)X(E,D)T motif of the I-Helix, known as the oxygen binding motif, which is part of a substrate recognition site 4 (Fig. 4). In CYP9Q P450s with high affinity for *N*-cyanoamidine neonicotinoids, including bumblebee CYP9Q6 and honeybee CYP9Q3, a serine is found at the normally highly conserved threonine position within the motif. Likewise, CYP9Q4, which metabolises both thiacloprid and acetamiprid, also diverges from the conserved threonine and has an alanine substitution. In contrast, other members of the CYP9Q family in *A. mellifera* (CYP9Q1 and CYP9Q2) and *B. terrestris* (CYP9Q5) which metabolise neonicotinoids with much lower efficiency, if at all, have the conserved threonine (Manjon et al., 2018). Further evidence indicative of the potential importance of the amino acid at this position comes from work on a neonicotinoid resistant strain of brown planthopper *Nilaparvata lugens* (Pang et al., 2016; Zimmer et al., 2018). In this example the CYP6ER1 P450 variant responsible for resistance to imidacloprid has a threonine to serine substitution; replication of this serine substitution in the wild-type enzyme resulted in a marked increase in the efficiency of imidacloprid detoxification (Zimmer et al., 2018). Finally, a threonine to proline substitution is found in an imidacloprid resistant strain of *Bemisia tabaci*, where overexpression of CYP6CM1 is linked with imidacloprid resistance (Karunker et al., 2008). Molecular docking and dynamic simulations of the interactions of imidacloprid with this enzyme revealed that Proline −326 forms part of the hydrophobic interface of the binding cavity and may stabilize binding of imidacloprid in the active site (Karunker et al., 2009).

Although the modification of the threonine residue within the oxygen binding motif appears to be important in allowing P450 enzymes to break down neonicotinoid compounds, it does not appear to be essential since other P450's associated with neonicotinoid metabolism, namely *Myzus persicae* CYP6CY3 (Bass et al., 2013) and *Drosophila melanogaster* CYP6G1 (Joussen et al., 2008), do not have equivalent substitutions at this position.

### 3.4 CYP9Q6 expression in transgenic *D. melanogaster*

To further confirm the role of CYP9Q6 in metabolising *N*-cyanoamidine neonicotinoids *in vivo*, we generated a transgenic line of *D. melanogaster* expressing *CYP9Q6* and examined its sensitivity to thiacloprid and acetamiprid. Expression of the transgene was confirmed via qPCR (Supplementary figure 4). In bioassays, flies expressing the CYP9Q6 transgene were significantly more resistant to both insecticides when compared to control flies of the same genetic background without the transgene (Table 1, Table 2). Flies expressing CYP9Q6 were approximately four times more resistant to thiacloprid (Table 1) and around 2.3 times more resistant to acetamiprid (Table 2). These results corroborate the metabolism assays with recombinant CYP9Q6 performed *in vitro* and demonstrate that this P450 can confer tolerance to thiacloprid and acetamiprid *in vivo*.

**Table 1:** Log-dose probit-mortality data for thiacloprid against *Drosophila melanogaster* CYP9Q6 and control strains.

Strain	Genotype	LD50 (mg/L)	95%CL	LD95 (mg/L)	95%CL	Resistance ratio (LD50)	Resistance ratio (LD95)
Control	*UAS::empty/* + *;Hsp::GAL4/*+	77.5	58.8–99.8	818.2	551.4–1402.6		
CYP9Q6	*UAS::CYP9Q6/*+*;Hsp::GAL4/*+	309	258–370	3326	2473–4768	3.99	4.06

**Table 2:** Log-dose probit-mortality data for acetamiprid against *Drosophila melanogaster* CYP9Q6 and control strains.

Strain	Genotype	LD50 (mg/L)	95%CL	LD95 (mg/L)	95%CL	Resistance ratio (LD50)	Resistance ratio (LD95)
Control	*UAS::empty/* + *;Hsp::GAL4/*+	9.3	8.37–10.32	23.79	20.6–28.34		
CYP9Q6	*UAS::CYP9Q6/* + *;Hsp::GAL4/*+	21.19	19.65–22.88	59.26	51.32–71.16	2.28	2.49

## 4 Conclusions

In summary, these data identify a novel CYP9Q P450 in *B. terrestris* that metabolises *N*-cyanoamidine neonicotinoids with high efficiency. This information, in combination with data on the previously characterised CYP9Q4 and CYP9Q5, can be used to identify and consequently avoid harmful pesticide effects on *B. terrestris* that may arise from the inadvertent inhibition of these enzymes (Mao et al., 2017). In addition, by adding to the number of known insect P450 enzymes that metabolise neonicotinoids, we highlight amino acids in the active site of P450s that may be important determinants of binding and metabolism of these compounds. This represents a useful starting point to further characterise the structure/function determinants of neonicotinoid metabolism using site-directed mutagenesis.

## Data availability

CYP9Q6 ORF and protein sequences were deposited on NCBI, accession no. MK559424.

## Conflicts of interest

Three authors of this study (M. Kohler, M. Zaworra, R. Nauen) are current employees of Bayer AG, manufacturer of neonicotinoid insecticides. The study was partially funded by Bayer AG.

## Appendix A Supplementary data

Data Profile Data Profile

Supplementary figures_amended_TGED_IB_2019_90 - v2 Supplementary figures_amended_TGED_IB_2019_90 - v2

CYP9Q6 manuscript_TGED_IB_2019_90 CYP9Q6 manuscript_TGED_IB_2019_90
