# Supplementary material for: Identification and functional characterisation of a novel N-cyanoamidine neonicotinoid metabolising cytochrome P450, CYP9Q6, from the buff-tailed bumblebee Bombus terrestris
Source: Insect Biochem Mol Biol. 2019 Aug;111:103171. doi: 10.1016/j.ibmb.2019.05.006 (PMC6675907; doi:10.1016/j.ibmb.2019.05.006)
Supplement: Supplementary figures_amended_TGED_IB_2019_90 - v2 [file mmc2.pptx]

## Slide 1
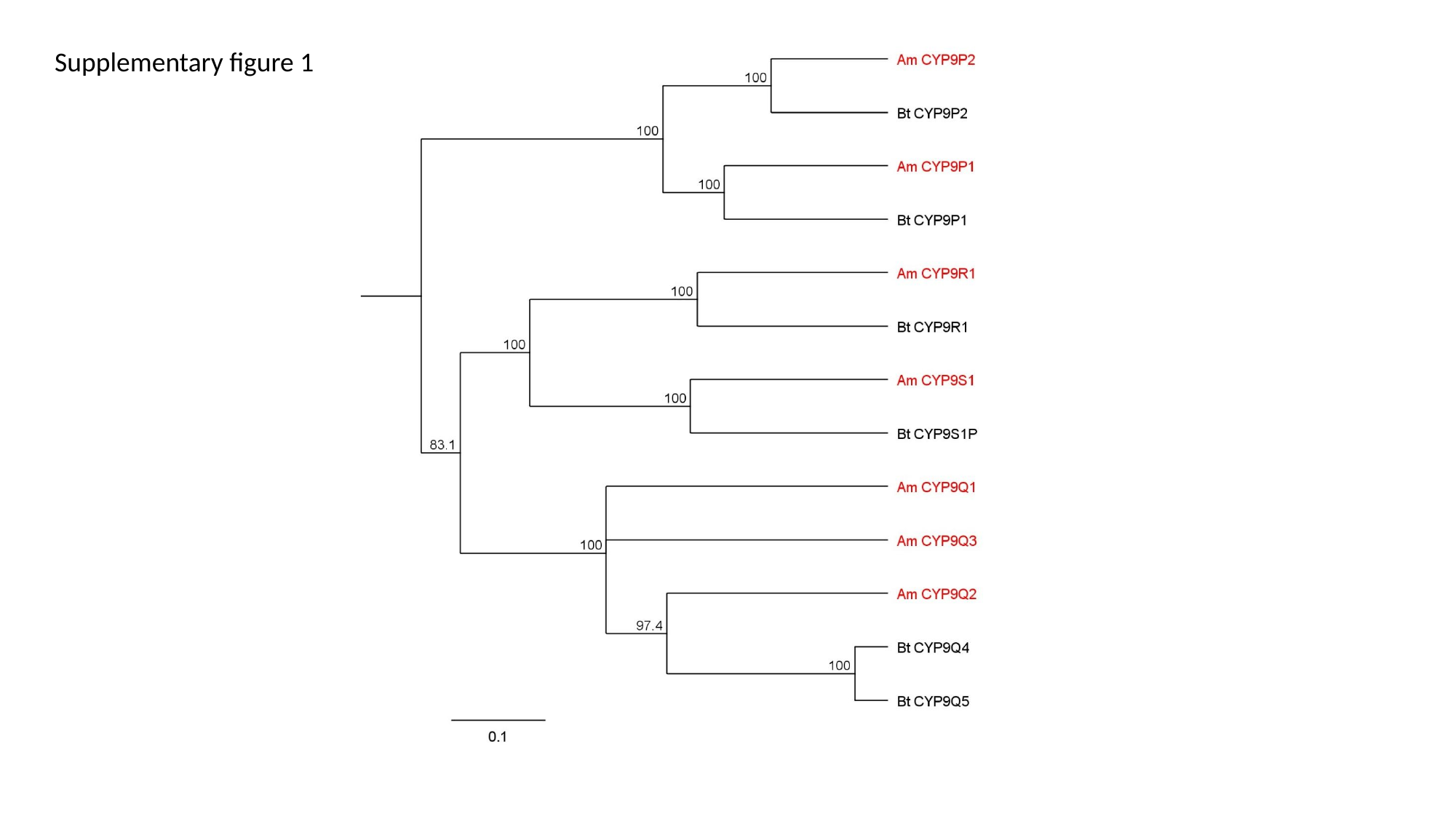

Supplementary figure 1

## Slide 2
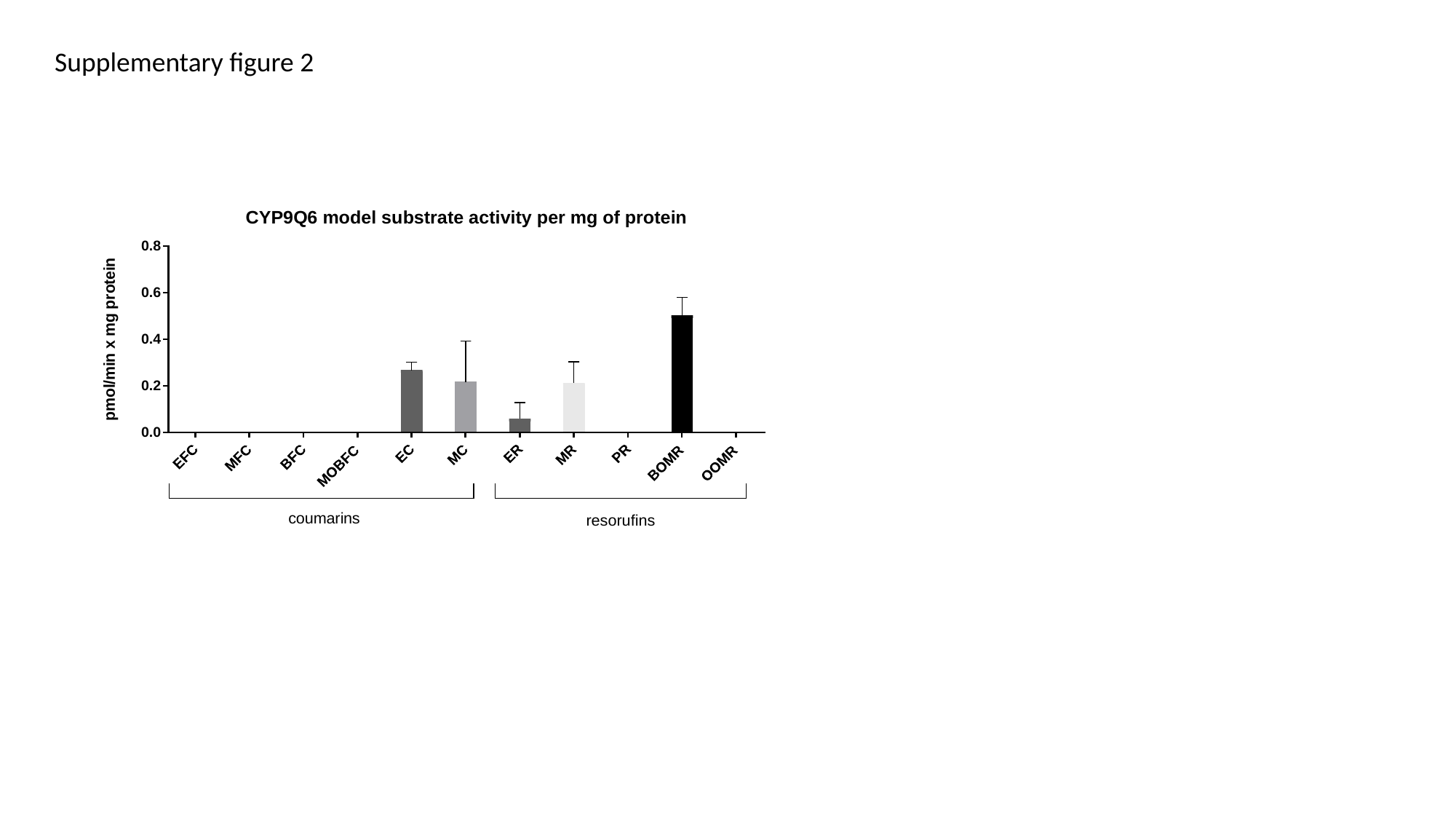

Supplementary figure 2

## Slide 3
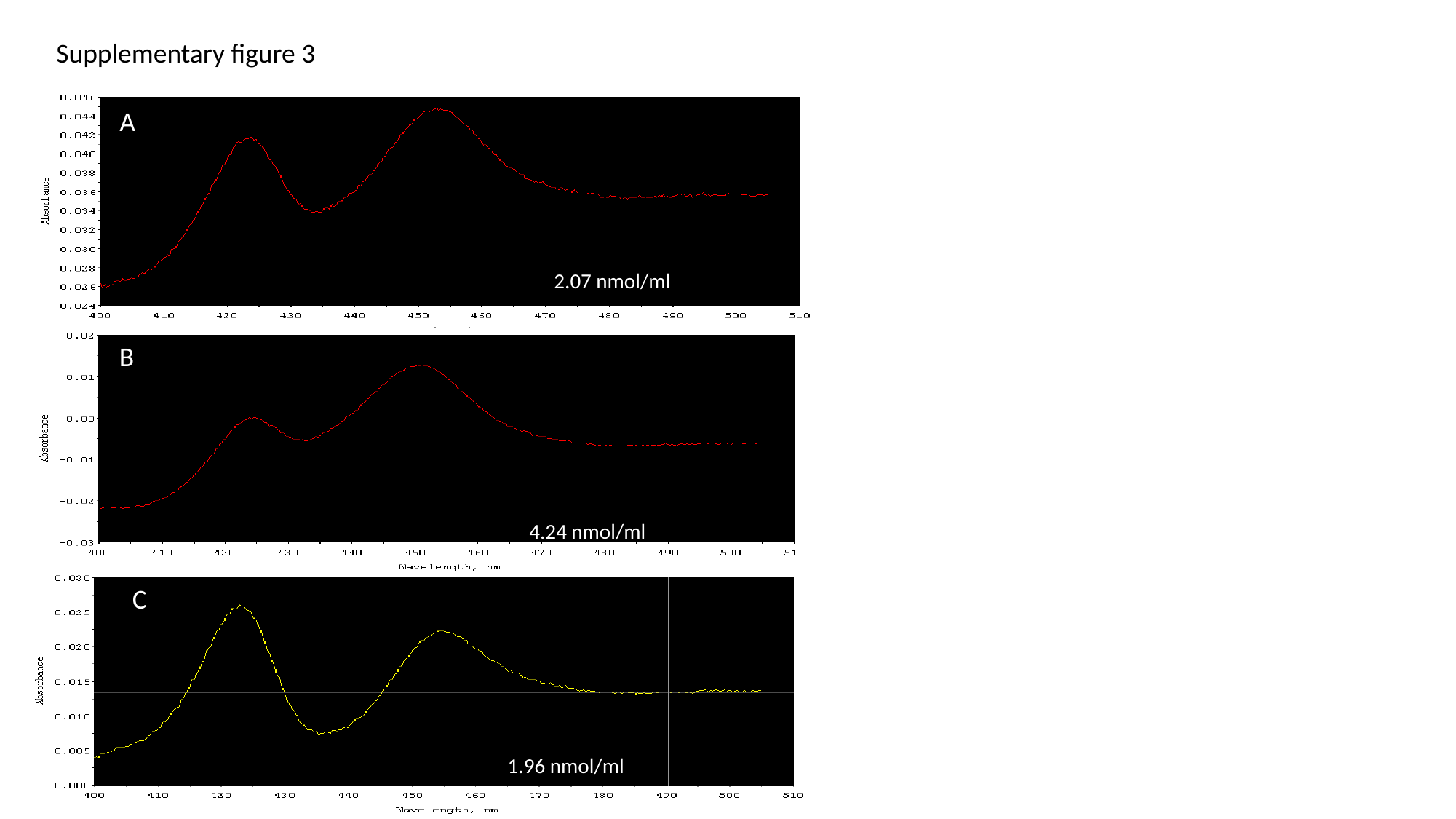

Supplementary figure 3
A
2.07 nmol/ml
B
4.24 nmol/ml
C
1.96 nmol/ml

## Slide 4
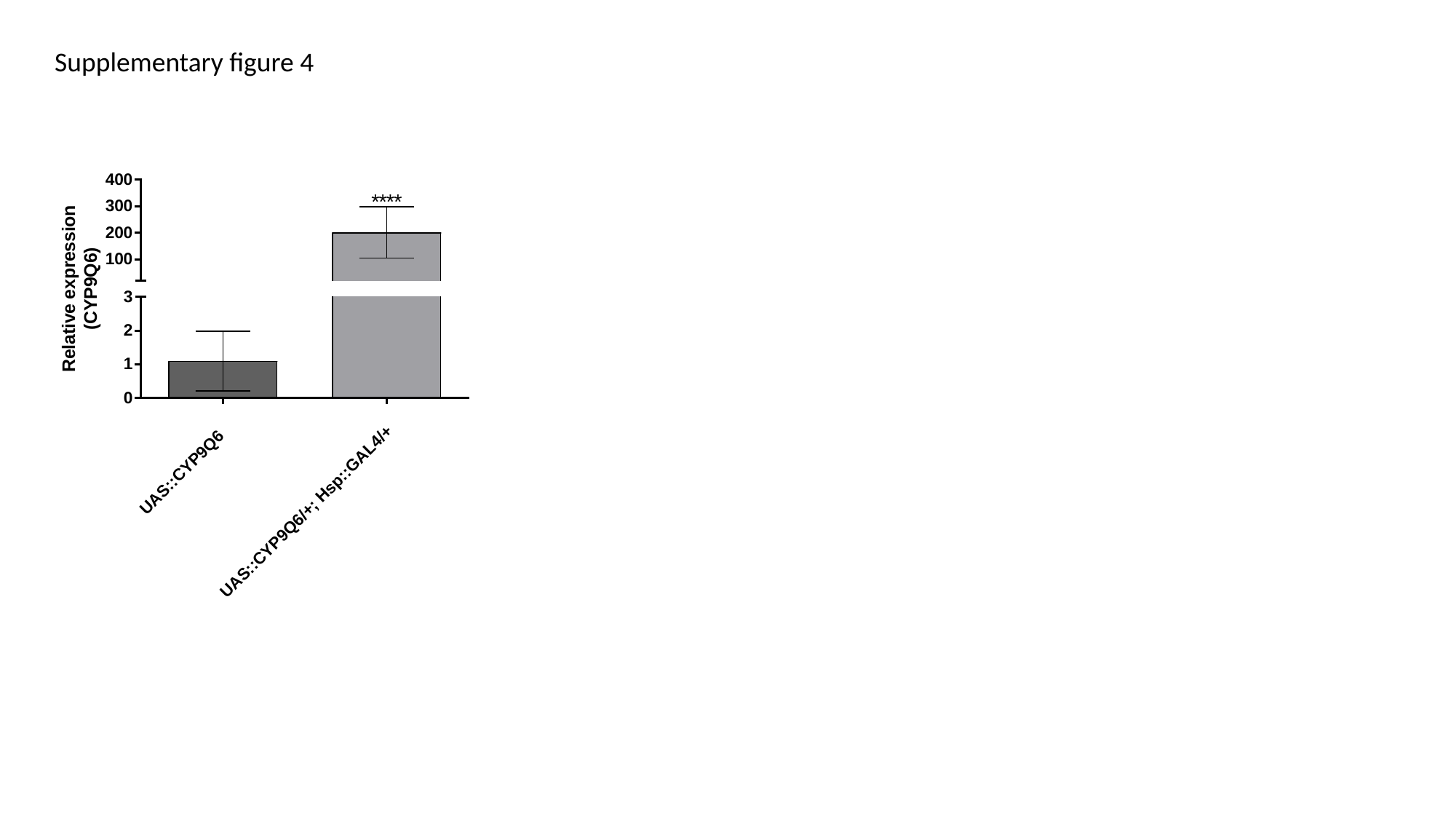

Supplementary figure 4
