# Supplementary material for: Identification and functional characterisation of a novel N-cyanoamidine neonicotinoid metabolising cytochrome P450, CYP9Q6, from the buff-tailed bumblebee Bombus terrestris
Source: Insect Biochem Mol Biol. 2019 Aug;111:103171. doi: 10.1016/j.ibmb.2019.05.006 (PMC6675907; doi:10.1016/j.ibmb.2019.05.006)
Supplement: CYP9Q6 manuscript_TGED_IB_2019_90 [file mmc3.docx]

.**Supplemental**

| **Primer name** | **Sequence 5’-3’** | **Used in amplifiation of:** |
| --- | --- | --- |
| **Bt RNA F** | AGTGGTTGTGGTATATTCGAATGG | RT-PCR CYP9Q6 ORF full length |
| **Bt RNA R** | CATTCGCAAATTCAATTTCTACAATTTCC | RT-PCR CYP9Q6 ORF full length |
| **Bt genomic F** | CGACACAGCAAACATCGTATCGG | gDNA PCR CYP9Q6 upsteam ORF |
| **Bt genomic R** | CTAGAAAACACTACTCCGTACGC | gDNA PCR CYP9Q6 downstream ORF |
| **Bt qPCR F** | GTGTGCTACCAGATACGCGA | qPCR- CYP9Q6 RNA forward |
| **Bt qPCR R** | AATGTTGGCGCAAAGGAACC | qPCR- CYP9Q6 RNA reverse |
| **PLA2 F** | GGTCACACCGAAACCAGATT | qPCR- phospholipase A2 forward |
| **PLA2R** | TCGCAACACTTCGTCATTTC | qPCR- phospholipase A2 reverse |
| **EEF1A F** | AGAATGGACAAACCCGTGAG | qPCR- elongation factor 1A forward |
| **EEF1A R** | CACAAATGCTACCGCAACAG | qPCR- elongation factor 1A reverse |
| **Q6 Q-PCR Fwd** | AATGTTGGCGCAAAGGAACC | qPCR- transgenic Drosophila, CYP9Q6 forward |
| **Q6 Q-PCR Rev** | CCTGGAACCTTCTTGTCGTT | qPCR- transgenic Drosophila, CYP9Q6 reverse |
| **F_qPCR_RpL32** | GCGCTTGTTCGATCCGTAAC | qPCR – transgenic Drosophila ribosomal protein L32 forward |
| **R_qPCR_RpL32** | GCCCAAGGGTATCGACAACA | qPCR – transgenic Drosophila ribosomal protein L32 reverse |
| **F_qPCR_sdha** | CACGACCCTCCATGATCTCG | qPCR – transgenic Drosophila succinate dehydrogenase subunit A forward |
| **R_qPCR_sdha** | CGGATGTCTCATCACCGAGG | qPCR transgenic Drosophila succinate dehydrogenase subunit A reverse |

**Supplementary table 1** PCR and qPCR primer sequences

**Supplementary figure 1.** Phylogenetic tree of annotated *CYP9* genes in *Apis mellifera* and *Bombus terrestris.*

**Supplementary figure 2**. Activity of CYP9Q6 against a range of coumarin model substrates. Error bars indicate Standard deviation, N=3. Abbreviations: EFC, 7-ethoxy-4-trifluoromethyl coumarin; MFC, 7-methoxy-4-trifluoromethyl coumarin; BFC, 7-benzyloxy-4-trifluoromethyl coumarin; MOBFC, 7-p-methoxy-benzyloxy-4-trifluoro coumarin; EC, 7-ethoxy coumarin; MC, 7-methoxycoumarin; ER, 7-Ethoxyresorufin; MR, 7- Methoxyresorufin; PR, 7-Pentoxyresorufin; BOMR, 7‐benzyloxymethoxy resorufin; OOMR, 7- octyloxymethylresorufin.

**Supplementary figure 3.** CO-difference spectra in reduced samples for A) CYP9Q4, B) CYP9Q5, C) CYP9Q6, together with active cytochrome P450 yields (nmol P450/ml).

**Supplementary figure 4.** Expression of recombinant CYP9Q6 transcripts in adult female *Drosophila melanogaster* crossed with a HSP-GAL4 driver line, relative to uncrossed parent. Significant difference in expression relative to the parent are indicated with ****P<0.0001 (two tailed T-test). N=4 group of 5 female adult flies. Error bars represent 95% confidence limits.
